# Submental abscess following anterior mandibular implantation: A case report and comprehensive literature review

**DOI:** 10.34172/japid.026.3991

**Published:** 2026-05-10

**Authors:** Elif Betul Yıldırım, Gulenay Colak

**Affiliations:** ^1^Department of Oral and Maxillofacial Surgery, Faculty of Dentistry, Gazi University, Ankara, Turkey; ^2^Department of Periodontology, Faculty of Dentistry, Gazi University, Ankara, Turkey

**Keywords:** Dental implants, Neck abscess, Peri-implantitis, Postoperative complications, Submental space

## Abstract

Dental implants are a predictable treatment for tooth loss; however, infections extending into deep fascial spaces are rare and potentially life-threatening. This case highlights the importance of early diagnosis and multidisciplinary management of an implant-related submental abscess in a diabetic patient. A 33-year-old female with type 2 diabetes mellitus (HbA1c: 8.1%) presented with submental swelling five months after anterior mandibular implant placement. Clinical examination revealed erythema, tenderness, and purulent discharge around implant #41. Cone-beam computed tomography (CBCT) showed complete resorption of the lingual cortical plate and a radiolucent tract extending into the submental space, indicating abscess formation. The implant system used was Megagen AnyOne® (4.0×10 mm). The patient was treated with amoxicillin–clavulanic acid 1 g every 12 hours for 5 days, and nonsteroidal anti-inflammatory drugs (NSAIDs) for pain control, followed by extraoral drainage with Penrose drain placement. The infected implant was removed surgically under local anesthesia, followed by thorough debridement of the cavity and local irrigation with rifamycin SV solution (250 mg/3 mL). The postoperative course was uneventful, and CBCT at the 3-month follow-up confirmed complete bone healing without recurrence. This case suggests that implant-related submental infections may result from a combination of surgical errors, anatomical factors such as lingual plate perforation, and systemic risk factors, including diabetes mellitus. CBCT plays a vital role in detecting cortical perforation and deep-space extension. Careful preoperative imaging, proper implant angulation, and timely combined medical-surgical management are critical for preventing serious complications and achieving favorable outcomes.

## Graphical Abstract

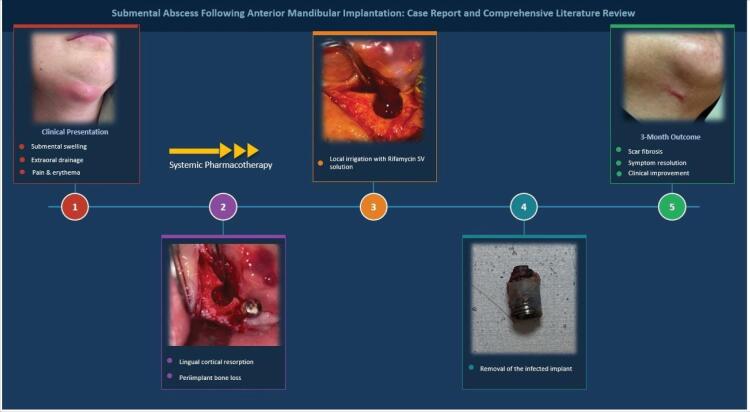

## Introduction

 Dental implants have become a widely used treatment for tooth loss and are recognized as a predictable, reliable option due to their high success rates. Previous studies have reported an average success rate of over 98% for implant placement and osseointegration.^1,2^

 Although the prognosis is generally favorable, complications associated with implant treatments may arise and, in some cases, can lead to serious consequences that may threaten life.^3,4^ In implant-related complications, various factors have been reported to play a role, including inadequate surgical or prosthetic treatment planning, traumatic surgical procedures, poor bone quality, microbial contamination, and irregular host inflammatory responses. These conditions can expose the implant to biological or mechanical complications, jeopardizing its long-term success.^5-7^ In this context, many researchers have examined the clinical and microscopic characteristics of implant-related periapical lesions. Complications reported include implant loss, sensory disturbances, soft tissue problems, peri-implantitis, progressive bone loss, and implant fractures.^8^ Among these, a particularly rare but clinically significant event is the extension of peri-implant infections into the perimandibular fascial spaces, resulting in abscess formation or extraoral fistula development.^9,10^

 Spread to the submental space usually occurs through extension from the submandibular or sublingual spaces, and less frequently, directly from the skin. Infections can spread rapidly between these interconnected fascial compartments, and if left untreated, a submental abscess can spread to adjacent spaces and progress to Ludwig’s angina. Ludwig’s angina is a rare, life-threatening clinical condition characterized by the rapid and phlegmonous infection of the bilateral submandibular, submental, and sublingual spaces.^11,12^ Furthermore, if the infection is not controlled, it may descend from the cervical spaces into the mediastinum, leading to another severe and potentially fatal complication such as necrotizing mediastinitis. Therefore, early diagnosis and accurate differential diagnosis are critical in preventing unnecessary interventions and ensuring timely implementation of appropriate treatment.^12^

 Although peri-implant infections extending to the submental region have been described in case reports, few studies have addressed the concurrent contribution of multiple factors that may facilitate their spread into deep fascial compartments. These factors include excessive lingual angulation, which can lead to lingual cortical perforation, and systemic conditions, such as diabetes mellitus, that can modify the host’s inflammatory response.

 This report presents a rare complication characterized by the development of an extraoral submental abscess before the formation of a fistula tract and skin drainage. This circumstance raises important clinical considerations, including the optimal timing for initiating advanced imaging, the appropriate threshold for proceeding to surgical drainage, and whether early implant removal should be contemplated to achieve effective source control. By documenting the decision-making process at this transitional stage, the present report provides a practical reference for clinicians who may encounter similar cases. To our knowledge, only a limited number of reports have radiographically documented both submental space involvement and lingual cortical perforation before fistula formation in cases of excessively lingualized anterior mandibular implants. The present case contributes to the existing literature by emphasizing two key clinical points: (1) the role of lingual cortical bone integrity in determining the direction of infection spread, and (2) the modifying influence of diabetes on the severity and progression of implant-associated deep fascial space infections. Additionally, this report synthesizes available evidence on mandibular implant-related infections with extraoral manifestations extending into the perimandibular fascial spaces.

## Case Report

**Figure 1:**
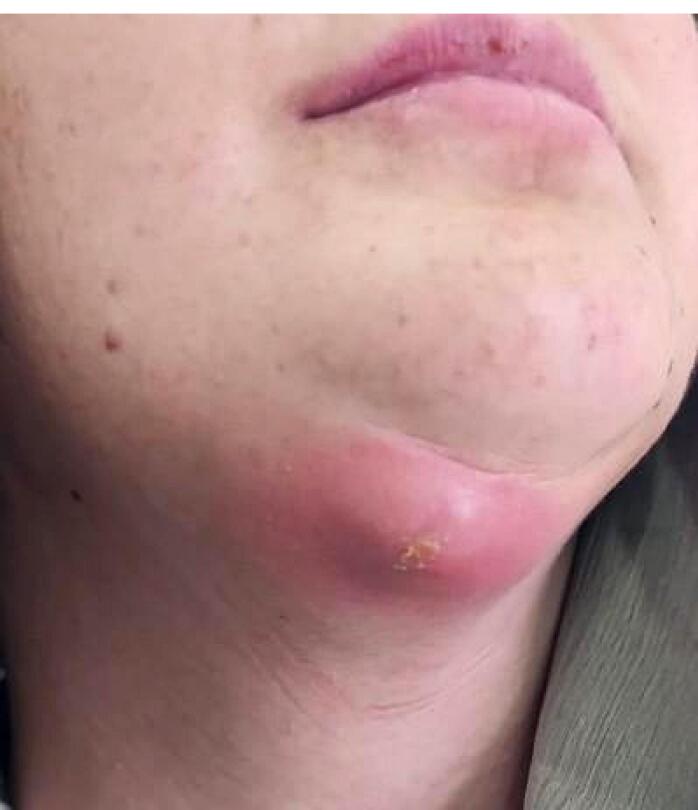
Extraoral photograph showing redness and a fluctuant swelling with a tense-elastic consistency in the anterior mandibular (submental) region, consistent with abscess formation before drainage

 This case report was prepared in accordance with the CARE guidelines. A 33-year-old female patient presented to the Gazi University Faculty of Dentistry Department of Oral and Maxillofacial Surgery clinic, complaining of swelling and pain on the skin surface of her lower jaw. Her medical history revealed that the complaints had emerged approximately five months after dental implants were placed. During the extraoral examination, redness and a fluctuating swelling of a tense-elastic consistency, warm to the touch, were detected in the anterior mandibular region (Figure 1). The patient’s systemic history included a diagnosis of diabetes mellitus (HbA1c: 8.1%), with current medical therapy consisting of Galvus Met 50/1000 mg, basal insulin glargine (Lantus 12 units/day), and insulin aspart (NovoRapid, 3 × 10 units/day). The intraoral examination revealed that the patient had three dental implants in the mandibular regions #31, #33, and #41.

 Periodontal evaluation revealed intense erythema around the implant in position #41, an 8-mm lingual periodontal pocket, and purulent discharge. The implants were clinically immobile; each withstood a 25-Ncm reverse torque test without evidence of mobility.

 To further evaluate potential etiological factors and determine the extent of the infection, panoramic radiography and CBCT (cone-beam computed tomography) were obtained.

**Figure 2:**
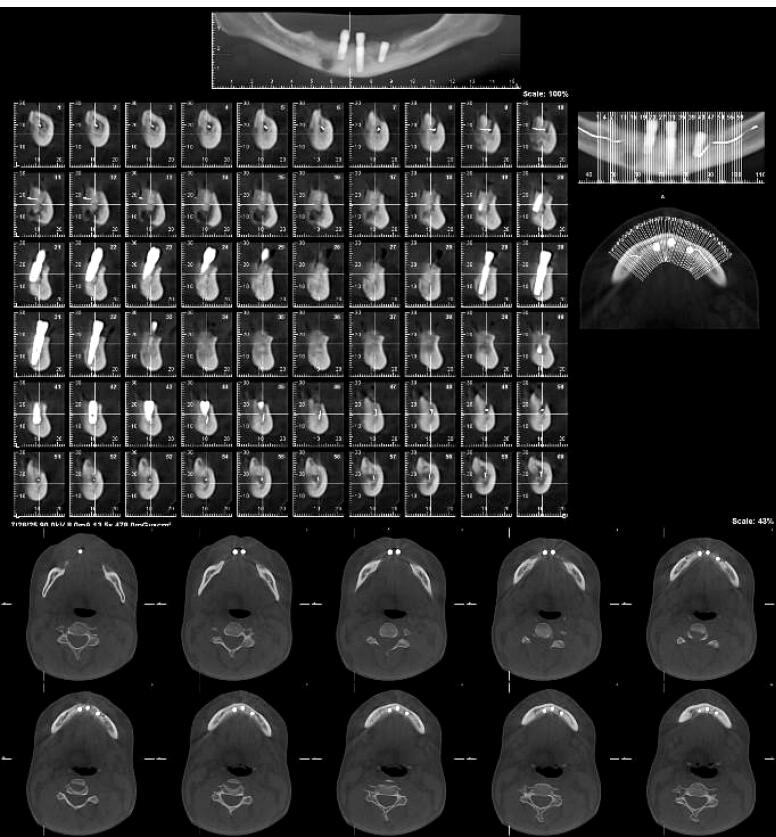
CBCT images showing the loss of lingual cortical bone continuity and a radiolucent area indicating resorption around the apical portion of implant 41 in the anterior mandibular region

 Radiographic examinations showed implants placed in regions #31, #33, and #41, with significant alveolar bone loss extending to the dental implants. According to the information obtained, the implant had been placed five months earlier in a private dental clinic. The implant used at the #41 site was a Megagen AnyOne® implant (MegaGen Implant Co., Ltd., Daegu, South Korea) with a diameter of 4.0 mm and a length of 10 mm. A poorly defined, destructive radiolucent lesion was observed in association with the apical region of the implant at the right mandibular central incisor site (#41). Increased perilesional sclerosis was also noted around the affected area. In the axial sections, loss of lingual cortical bone integrity was evident, particularly in the apical region of implant #41 (Figure 2). This significant resorption suggests the potential for infection to progress into the submental space. When evaluated in conjunction with clinical findings, the radiographic appearance supported the presence of an extraoral submental abscess.

 Based on the clinical and radiographic assessment, chronic osteomyelitis secondary to implant infection was considered in the differential diagnosis. Consequently, after comprehensive evaluations, a decision was made to remove the implants. Written informed consent was obtained from the patient for publishing this case report and the accompanying clinical images.

**Figure 3:**
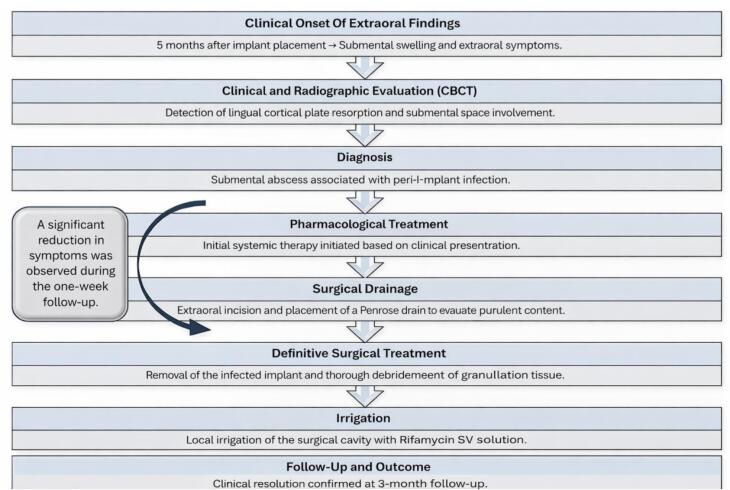
Clinical flowchart summarizing diagnosis, staged management, and follow-up of implant-related submental abscess

Figure 3 presents the chronological progression of diagnosis and management.

###  Treatment Approach

 The treatment procedure involved both extraoral and intraoral approaches. Following diagnosis, the patient was prescribed amoxicillin/clavulanic acid 1 g orally every 12 hours for 5 days (Augmentin®, GlaxoSmithKline, Brentford, UK). Nonsteroidal anti-inflammatory drugs (NSAIDs) were also recommended for postoperative pain management under medical supervision.

###  Abscess Drainage

 Based on the history and clinical findings, drainage of the abscess was planned first. Drainage was achieved by making an incision with a #12 scalpel in the extraoral swelling area. Subsequently, manual debridement was performed in the affected fascial spaces, and a large amount of purulent exudate was drained. A quarter-inch Penrose drain was placed and secured with a 5-0 Prolene suture, and the patient was scheduled for daily follow-up visits over one week. A significant reduction in the patient’s pain was observed at the follow-up appointment one week later. Based on clinical and radiographic findings, the infection source was determined to be implant #41, which was removed.

###  Surgical Phase 

**Figure 4:**
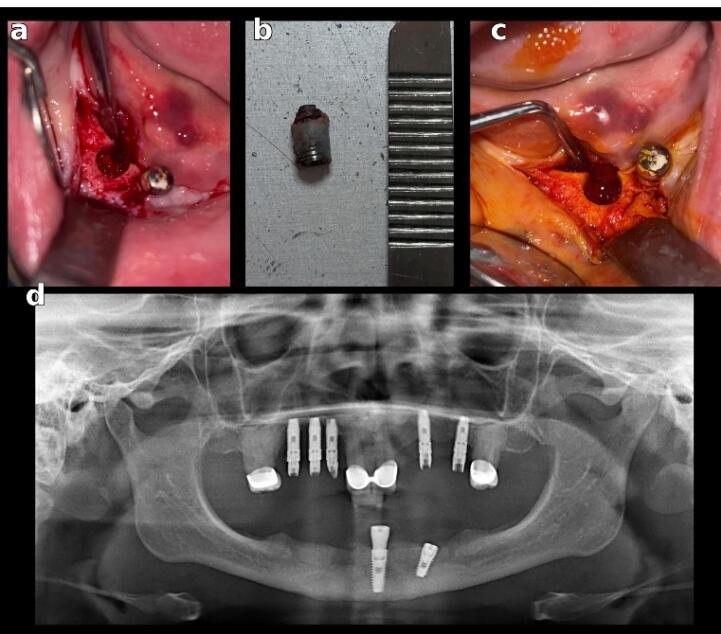
Intraoperative and postoperative findings. (A) Intraoral view showing complete resorption of the lingual cortical plate after flap elevation. (B) Appearance of the removed implant. (C) Cavity debridement and irrigation performed with Rifamycin SV solution (250 mg/3 mL). (D) The panoramic radiograph obtained at the third postoperative month demonstrates the resolution of the infection and satisfactory healing of the surgical site

 Local anesthesia in the anterior mandibular region was achieved using Ultracaine Fort (Articaine HCl 40 mg/mL + Epinephrine HCl 0.0125 mg/mL, Sanofi, Turkey). When the flap was elevated, complete resorption of the lingual cortical plate was observed at implant #41 (Figure 4A). The implant was removed, and the granulation tissue within the cavity was thoroughly debrided (Figure 4B). The area was then irrigated with a Rifamycin SV solution (250 mg/3 mL ampoule) (Figure 4C). The surgical site was primarily closed with 4-0 non-resorbable sutures. During the first postoperative week, the sutures were removed, and the site demonstrated satisfactory healing. At the three-month follow-up, no signs of infection or fistula formation were observed.

###  Follow-up and Outcomes

**Figure 5:**
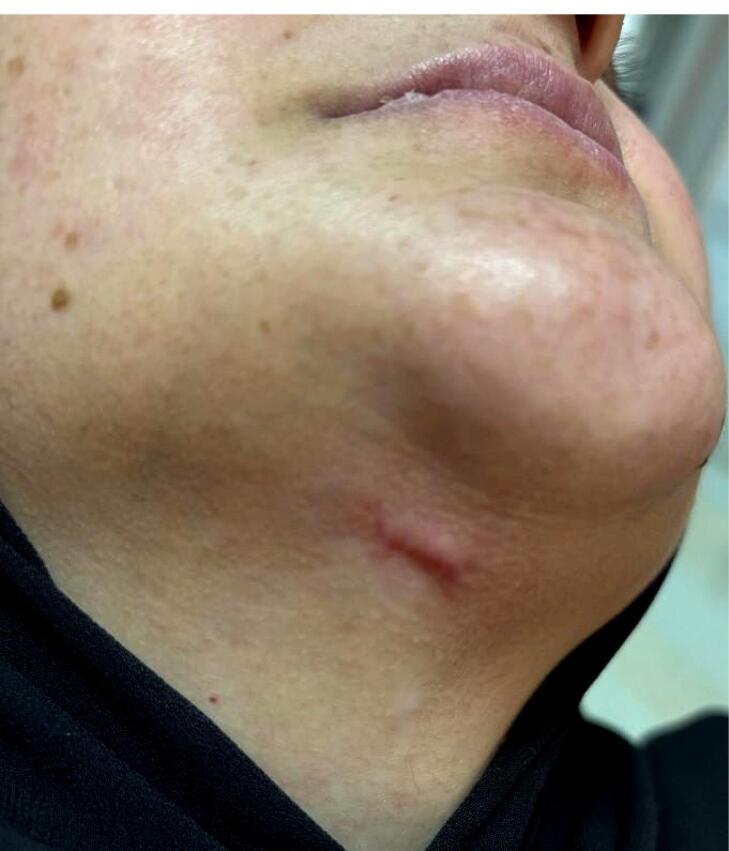
Extraoral clinical view at the 3-month follow-up demonstrating complete resolution of infection. No erythema, swelling, or drainage is present. Fibrotic scar tissue is observed along the incision line in the submental region

 The patient was clinically monitored weekly during the first postoperative month and at 3 months. Progressive reduction in extraoral swelling, pain, and erythema was observed within the first week following abscess drainage. The patient expressed satisfaction with the treatment outcome and appreciation for the rapid recovery. At the 1-month follow-up, intraoral soft tissue healing was satisfactory, with no purulent discharge or recurrence of swelling. CBCT evaluation at the 3-month follow-up demonstrated radiographic evidence of bone fill, with no findings suggestive of persistent cortical perforation or deep space involvement (Figure 4D). The surgical site remained infection-free, and no further complications were observed (Figure 5). The patient has not yet undergone final prosthetic rehabilitation; this will be planned following complete ridge remodeling.

## Discussion

 To date, reports describing radiographic identification of submental space involvement and lingual cortical perforation before fistula formation in excessively lingualized anterior mandibular implants remain limited.

 Today, dental implants are considered biomaterials with high success rates for the functional and aesthetic rehabilitation of missing teeth.^13^ Despite the generally favorable prognosis of dental implants, certain risk factors, including inadequate preoperative planning, the presence of oral infections, systemic diseases, and dysregulated host inflammatory mechanisms, may lead to complications. When these occur, they can have severe clinical consequences, such as the onset of peri-implantitis, systemic spread of infection, progressive implant loosening, and ultimately, implant loss.^14-17^ This case is clinically significant in that the mandibular lingual bone was completely resorbed five months after implant placement, leading to the development of an extraoral abscess. In our patient, the rapid bone loss and abscess formation are thought to have resulted from the interaction of multiple factors.

 The ideal implant position is critical for long-term success. However, due to anatomical limitations, it may not always be possible to place the implant in the desired position. Although the mandibular anterior region is considered a relatively safe area for dental implant placement, lingual crest perforations can occur there. According to the literature, perforation of the lingual cortex during implant placement is one of the most common factors leading to the formation of an infection pathway between the oral cavity and deep tissues. Particularly in cases such as ours, lingual concavity and inclination during osteotomy in the edentulous mandibular anterior region increase the risk of perforation of the lingual cortical plate.^12,18^ In this case, incorrect implant angulation during placement may have caused perforation of the lingual cortex. Such a perforation may allow oral microorganisms to invade the lingual area, thereby paving the way for the development of peri-implant infection.^19^

 Furthermore, the implant in this case was placed with excessive lingual inclination, which likely increased mechanical pressure on the thin lingual cortical plate and facilitated its resorption. This positional error appears to have been a decisive factor in the development and downward spread of infection into the submental space. While lingual perforation has been discussed in previous reports, the present case highlights that excessive lingualized implant positioning itself can be an initiating factor for cortical compromise and the spread of infection—a point not explicitly documented in earlier literature. Thin cortical structures are prone to ischemia and subsequent resorption due to insufficient vascularization. Moreover, the anterior mandibular region, characterized by reduced blood supply, has been associated with the highest rates of early implant failure.^17,18,20^ Preoperative computed tomography should be used to assess mandibular anatomy and identify possible anatomical variations during implant planning in the anterior mandible.^12^

 Although dental implant placement can be performed in diabetic individuals, previous studies have reported a higher incidence of clinical complications in this population.^21,22^ Among systemic conditions, diabetes mellitus has long been recognized as a significant risk factor in implant therapy due to its adverse effects on soft and hard tissue healing.^23^ In patients with diabetes, accelerated osteoclastogenesis and increased bone resorption have been reported, which may adversely affect bone remodeling and compromise osseointegration.^24^ Dahihandekar et al.^25^ evaluated bone mineral density (BMD) at prospective mandibular implant sites in patients with and without type 2 diabetes using CBCT, and reported that individuals with type 2 diabetes exhibit significantly reduced BMD in the lingual cortical plate and trabecular region, whereas the buccal cortical plate appears to remain unaffected.^25^ This finding supports the pattern of lingual bone resorption observed in our case, suggesting that the lingual cortical bone may be structurally weaker in diabetic individuals and therefore more prone to resorptive changes following implant therapy.

 The issue of whether the surgical site and implant surface were protected from possible exogenous contamination during surgery should not be overlooked.^26,27^ The findings in our case also suggest that the implants may have been placed immediately into an area that had not undergone adequate alveolar debridement and decontamination and may have been exposed to pre-existing bacterial contamination. However, when appropriate debridement and decontamination protocols are applied, immediate implant placement in infected extraction sockets is not considered an absolute contraindication. The literature reports positive clinical outcomes for implants placed in infected extraction sockets following effective debridement.^28^ Nevertheless, case selection must be carefully performed, areas with active suppuration must be avoided, and perioperative infection control must be prioritized.^27^

 When all these factors are considered together, it can be concluded that the complications in our patients have a multifactorial etiology; they may stem from errors in the surgical technique or from factors related to the patient’s systemic condition. This case documents the pre-fistulization stage of a submental abscess secondary to implant-related infection, confirmed radiographically by CBCT. It demonstrates how early CBCT-based diagnosis can reveal lingual cortical perforation and submental space involvement before overt clinical fistula formation—a presentation rarely documented in the literature. When a submental abscess is detected or there is suspicion of a deep neck infection, it is crucial to closely monitor the patient, perform imaging to determine the extent of spread, and immediately initiate an appropriate treatment plan.^12^

 Early diagnosis and timely intervention are critical in emergency airway management, intravenous antibiotic treatment, and surgical drainage in the presence of abscesses.^29^ While cellulitis and small abscesses frequently resolve with antibiotics alone, operative drainage should be pursued in the presence of large abscesses, Ludwig’s angina, anterior visceral space involvement, or antibiotic nonresponse.^30^ In treating infected implants, it has been reported that preserving the implant should be prioritized when there is no acute infection, and antibiotic treatment should be recommended as the first-line treatment. If the infection persists, surgical debridement may be necessary if there is evidence that implant preservation is possible.^14^

 In the management of maxillofacial abscesses, particularly in cases with a high risk of spread, initiating empirical broad-spectrum antibiotic therapy is a commonly adopted approach.^31^ The selection of an appropriate antibiotic regimen plays an important role in the clinical course of maxillofacial infections. Although bacterial culture and antibiotic susceptibility testing provide valuable guidance, obtaining these results typically takes several days; therefore, empirical antibiotic therapy is often initiated while awaiting test results. However, if the initial empirical therapy does not yield adequate clinical improvement, treatment should be re-evaluated and modified based on the microbiological profile and susceptibility results.^32^ In our case, a reduction in clinical symptoms was observed following the initial empirical antibiotic therapy; therefore, the administered antibiotic protocol was considered to have provided an adequate clinical response.

 Rifamycin exhibits antibacterial activity against both Gram-positive and Gram-negative bacteria. In dentistry, it has been used locally to irrigate infected sites, such as fistulas, sinuses, abscesses, osteomyelitic areas, and root canals. Its topical application on bone tissue has been associated with a reduced incidence of infectious complications.^33^ Clinical and experimental studies have further demonstrated that rifamycin irrigation supports local infection control and has been shown to reduce alveolitis incidence, prevent postoperative infection, and provide effective analgesia.^34,35^ In the present case, rifamycin was applied following implant removal to help achieve effective local infection control.

 To contextualize the present findings within the existing body of evidence, a literature search was conducted to identify previously reported cases of implant-associated infections extending into the perimandibular fascial spaces. PubMed and Google Scholar databases were searched up to September 2025 using the keywords “dental implant,” “perimandibular abscess,” “submental abscess,” “submandibular space infection,” “orocutaneous fistula,” and “deep neck space infection” in various combinations. Only case reports and case series describing infections associated with mandibular dental implants that demonstrated abscess formation or fistula development involving the submental, submandibular, or sublingual spaces were included. Publications unrelated to dental implants or lacking clinical and radiographic evidence of perimandibular space involvement were excluded.

 The sublingual, submental, and submandibular spaces are anatomically interconnected, and the term ‘perimandibular spaces’ is used to describe them collectively. This close anatomical continuity facilitates the rapid spread of infections into these compartments.^11,36^ The sublingual and submental spaces are adjacent fascial compartments separated by the mylohyoid muscle, and infections may spread around this muscular barrier. In general, infections originating in the anterior mandible remain confined to the sublingual space when they arise above the mylohyoid line, whereas those that extend below or around the muscle can spread into the submandibular or submental spaces. Submental abscesses typically do not originate directly from the apex of a tooth or implant, but rather result from the progression of infection from the sublingual or submandibular spaces.^11^ In the present case, perforation of the lingual cortex likely allowed purulent material to initially enter the sublingual space and then extend inferiorly around the mylohyoid muscle into the submental space, presenting clinically as swelling beneath the chin. If left untreated, such infections may rapidly progress to involve the bilateral submandibular spaces or extend along the deep cervical fascia into the mediastinum.^12^ Early imaging and timely drainage in this case prevented these severe complications.

Table 1 summarizes the current literature on cases in which abscesses or fistulas developed in the perimandibular spaces. In the reviewed cases, implant removal has been reported as the final treatment option in most advanced presentations with extraoral findings. In our case, antibiotic treatment and surgical drainage were initiated immediately after the diagnosis of the submental abscess. However, clinical and radiographic examination revealed that the lingual cortical wall of the implant in the mandibular anterior region was wholly lost, and there was severe bone destruction. Considering these anatomical conditions, limited intervention was deemed insufficient. Considering the possibility of the infection spreading to the deep cervical fascial spaces, removing the implant was considered the most appropriate treatment strategy.

**Table 1:** Review of case reports on mandibular abscess or fistula formation with a focus on the perimandibular spaces

**Author and year**	**Infection site/ spread**	**Predisposing factor(s)**	**Treatment**	**Outcome**
Nkenke et al.^37^ (2004)	submental space (cutaneous fistula)	history of oral SCC resection + peri-implantitis	extraoral excision of sinus tract + implant removal	complete resolution
Markiewicz et al.^38^ (2007)	buccal mandibular space (orocutaneous fistula)	subperiosteal implant failure (cobalt alloy-associated corrosion)	implant removal + fistula tract excision + systemic antibiotics	complete resolution
Silva et al.^27^ (2010)	submental space (suppurative fistula)	immediate implant in an infected site	explantation + curettage	complete resolution
Cariati et al.^39^ (2019)	submental + bilateral cervical abscess + anterior mediastinum	lingual cortical perforation with implant instability	implant removal + IV antibiotics + steroids + NSAIDs	complete resolution
Cillo & Barbosa^40^ (2019)	submental + submandibular spaces	immunosuppression (Adalimumab therapy)	extraoral incision and drainage + removal of all implants + antibiotics	complete resolution
Cardoso et al.^9^ (2020)	submental + sublingual + submandibular (Ludwig’s angina)	displacement of the implant into the submandibular space	extraoral implant removal + drainage + IV antibiotics	complete resolution
Elwany et al.^41^ (2024)	submental + sublingual + bilateral submandibular + pretracheal spaces	lingual cortical perforation with implant instability”	surgical drainage + implant removal + culture-guided IV antibiotics	complete resolution
D’Angeli et al.^12^ (2025)	submental + submandibular spaces	peri-implantitis-associated infection	surgical drainage + implant removal + systemic antibiotics	complete resolution

Abbreviations: SCC = squamous cell carcinoma; IV = intravenous; NSAIDs = non-steroidal anti-inflammatory drugs

 Across the cases presented in Table 1, hospitalization is frequently indicated when pain and facial swelling are accompanied by fever, dysphagia, dyspnea, and/or hoarseness, given the potential for rapid spread and airway compromise. These patients should be closely monitored and administered intravenous broad-spectrum antibiotics, with emergency airway management and surgical drainage planned if necessary.^9,41^ In contrast, as no findings supporting systemic toxicity or airway compromise were identified in the presented case, oral antibiotic therapy under outpatient observation was deemed appropriate. Based on the cases summarized in the presented literature, abscesses extending into the perimandibular spaces most frequently occurred in association with dental implants placed in the interforaminal region of the anterior mandible, where anatomical vulnerability of the lingual cortex is well recognized. In a substantial number of reports, implant removal was ultimately required as the definitive treatment. This pattern indicates that, once extraoral abscess formation or cutaneous fistulization has occurred, conservative debridement alone is often insufficient to achieve infection control, thereby necessitating a combined approach involving surgical drainage and implant explantation. Collectively, these findings underscore the importance of recognizing the anterior mandible as a high-risk zone for deep fascial spread in the event of peri-implant infections, and they highlight the need for prompt intervention to prevent progression to more severe cervical space involvement.

## Conclusion

 This case underscores the critical role of CBCT in the early detection of implant-related infections extending into deep fascial spaces that may not be visible on conventional radiographs. Particular attention to implant angulation and lingual cortical bone integrity in the anterior mandible is essential, as structural compromise may facilitate the spread of infection into adjacent compartments, such as the submental space. Although rare, such infections may progress rapidly and lead to severe complications, especially in patients with systemic conditions such as diabetes mellitus. Therefore, meticulous preoperative planning, precise three-dimensional implant positioning, strict aseptic technique, and timely combined medical–surgical management are crucial to minimize risk and prevent potentially life-threatening outcomes.

## Competing Interests

 The authors declare that they have no competing interests regarding authorship and/or publications of this paper.

## Consent for Publication

 Written informed consent was obtained from the patient for the publication of this case and the accompanying clinical images.

## Data Availability

 All data generated or analyzed during this study are included in this published article.

## Ethical Approval

 Not applicable.
